# Operating Department Practitioner’s research priorities: A Delphi study

**DOI:** 10.1177/17504589251330423

**Published:** 2025-04-22

**Authors:** Adele Nightingale, Victoria Cadman, Victoria McIntyre, Serene Pachniuk, Harrison Murdoch

**Affiliations:** 1 University of Bolton, Bolton, UK; 2 Sheffield Hallam University, Sheffield, UK; 3 Royal Hallamshire Hospital, Sheffield, UK

**Keywords:** Operating Department Practitioners, ODP, Research priorities, Research capacity, Research culture

## Abstract

With the 2022 Allied Health Professions’ Research and Innovation Strategy and the College of Operating Department Practitioners joining the Council for Allied Health Professions Research, understanding the Operating Department Practitioner profession’s vision for research and innovation and identifying its research priorities has become important. This Delphi study aimed to establish research priorities for the Operating Department Practitioner profession. Questionnaires were distributed to Operating Department Practitioners using College of Operating Department Practitioners and social media networks. Round 1 saw 49 eligible responses; this reduced to 21 in Round 2 and 17 in Round 3. Thirty-one research priorities were identified by consensus. Priority rank was determined by mean score, percentage agreement, and coefficient of variance. By reaching a consensus, Operating Department Practitioners co-created research priorities and identified several themes that will contribute to professional development and patient care and support funding opportunities. The five key themes were Workforce Transformation, Education, Patient Safety and Experience, Innovation and Technology, and Theatre Culture.

## Introduction

With the Allied Health Professions’ Research and Innovation Strategy, published in 2022 (Health Education England (HEE) 2022), and the College of Operating Department Practitioners (CODP) joining the Committee (formerly Council) for Allied Health Professions Research (CAHPR), there is an evident need to understand the vision of the Operating Department Practitioner (ODP) profession regarding research and innovation. It is important to appreciate how ODP(s) would contribute to the national and global agendas, which the Allied Health Professions (AHP) strategy aims to influence. To proactively contribute to the profession, we must understand its current research priorities. To address this issue, we designed and conducted a modified Delphi Study. This project aims to establish consensus among ODPs on priority areas for future ODP research.

## Background

Despite ODPs being registered to and regulated by the Health and Care Professions Council (HCPC) for 20 years, published research related to and undertaken by ODPs remains lacking (Nightingale 2019). This leaves gaps in the development of new knowledge, skills, and professional development of ODPs.

Research is distinctly embedded within the CODP Curriculum (2018), which explicitly details the expectation that the ODP programme leads should ‘actively engage in research and/or contribute to professional body of knowledge via publication’ (CODP 2018: 11). The curricula also detail an expectation for students to undertake activities that ‘provide a focus of personal research’ (CODP 2018: 14). This places research as an important part of the ODP curriculum. In addition, the HCPC (2023), Standard of Proficiency for ODP, has research threaded throughout, especially regarding Section 13, ‘drawing on appropriate knowledge and skills to inform practice’ (p. 15). These two key documents guiding ODPs’ professional development mark research as a skill required throughout an ODP’s career.

Currently, the CODP does not have a research strategy, even though it aligns with the Allied Health Professionals (AHP) Research and Innovation Strategy (HEE 2022). The latter is pivotal in driving the ODP research agenda. The strategy clearly articulates a vision that is the collective vision of all AHP professions but has the fluidity to address the unique needs of individual professions. This strategy aims to address the growth, stability, and sustainability of research and innovation within AHP professions (HEE 2022). Furthermore, NHS England has developed a Multi-professional Practice-based Research Capabilities Framework (NHS England 2024) to promote the active engagement of health and care practitioners to ensure safe evidence-based practice. It is designed to strengthen and develop the evidence base and inform service design, clinical reasoning, and shared decision-making with people and communities. Boaz et al (2015) highlighted the importance of active research engagement through their work, demonstrating an improvement in healthcare performance when clinicians and healthcare organisations are engaged in research. Such engagement is emphasised by the four pillars of practice and has also been identified as an area of need within the National Operating Department Practitioner Workforce Programme Report (HEE 2023). It also relates to the NHS People Plan on utilising the full range of staff skills and experience to optimise outcomes and experience (NHS England 2020).

For ODPs, this drive initiated professional conversations between the CODP and the authors of this article with respect to understanding research priorities. Having a greater understanding of research priorities for ODP will support the profession in clearly aligning itself with the AHP strategy. It will also give the profession a clear vision with the ability to influence agendas, including patient safety, education, and professional development.

## Methods

A modified Delphi method was employed to establish research priorities for the ODP profession by offering respondents the opportunity to indicate their individual opinions and allowing analysis to establish a consensus. Although there are no set criteria for modified Delphi studies, key components identified as good practices include participant descriptions, setting threshold criteria for consensus, and doing so a priori, which were integrated into our design (Diamond et al 2014, Foth et al 2016, Nasa et al 2021). There are also no set reporting guidelines for the Delphi method; however, the ACCORD checklist has been utilised to support the reporting of this study and assess its strengths and limitations (Gattrell et al 2024). Delphi consensus methods have previously been utilised by others to establish priorities within individual professions (Porter et al 2020, Rankin et al 2012, Rushton et al 2014, Society and College of Radiographers (SCoR) 2017) and clinical specialities (Jarman et al 2023, Kelly et al 2018, McElroy et al 2022, Ramage et al 2023). Such practicalities were also applicable for ODPs, facilitating input from all specialisms and areas of practice and removing the barriers and constraints of holding in-person meetings and/or workshops to establish priorities. Given that this was the first priority-setting project and the low representation of ODPs within the research field (HEE 2023), it was deemed important to include all ODPs, including students, with an interest in research. This will hopefully encourage them to have a voice in shaping the future of research for the profession. As such, a specific ‘expert’ panel was not formed. This approach was successfully adopted by osteopaths in the UK (Rushton et al 2014). Participants were recruited through advertising via CODP, social media, and circulation through ODP networks. The recruitment flyer contained a QR code linked to Round 1. It contained hyperlinked participant information sheets about the entire study along with further details on Round 1. Consent to participate was obtained from the first section of the electronic form. Due to the quasi-anonymous nature of Delphi Studies, the final part of the form requested the participants to provide their e-mail addresses if they were happy to participate in the next round of the study. This was repeated in each round, with responses being anonymised from the provided e-mail addresses before analysis. Data were stored on secure drives at the researchers’ institutions in line with ethical approval and GDPR guidance. Ethical approval was granted by the University of Bolton (UREC0055) and registered with Sheffield Hallam University (ER64511526).

### Round 1

Round 1 was open to responses for 2 weeks. Participants’ demographic information was collected to understand the representation of various levels of education, job roles, and geographical areas. A single question formed the main part of the Delphi study; ‘Please list up to 3 areas which you believe are a priority for ODP research.’ This provided flexibility to include a wide range of practices while prioritising maintaining focus rather than becoming an extensive, open-ended question. Two researchers reviewed the anonymised responses independently to code and merge similar responses into provisional themes. They then resolved any identified issues and reached an agreement on what was to be presented in Round 2.

### Round 2

Round 2 was circulated via e-mail to all participants who had provided their e-mail addresses with consent; the link was open for 2 weeks. Round 2 comprised a list of the identified priorities grouped into provisional themes. It utilised a Likert-type scale (Strongly Disagree to Strongly Agree), where participants scored how much they agreed/disagreed with each research priority. Before requesting participation in Round 3, participants were asked whether any priorities were missing from the list they felt deserved consideration and to provide details for them. The responses were converted to a numerical format (1 = strongly disagree to 5 = strongly agree) for analysis. An a priori threshold for consensus was achieved when the following three criteria were met:

- A mean rating of ⩾ 4.0
- A coefficient of variation (CV) of ⩽ 30%
- ⩾75% agreement (% of participants scoring 4 = Agree or 5 = Strongly Agree.

These criteria reflect those used by other UK-based AHPs in priority-setting studies (Rankin et al 2012, Rushton et al 2014, ScoR 2017) and follow good practice. This includes setting threshold criteria, doing so a priori, and utilising a 75% agreement. While there is extensive variance in the value used for percentage agreement, two extensive systematic reviews of Delphi studies identified 75% as the median threshold agreement utilised (Diamond et al 2014, Foth et al 2016). Priorities meeting all predefined threshold criteria were deemed as meeting consensus, and therefore, a research priority for the ODP profession. Free text responses regarding topics the participants felt were missing from the list presented in Round 2 were reviewed using the same approach as in Round 1.

### Round 3

Round 3 presented additional priorities identified from the open question in Round 2. This round was circulated only to those who responded and provided a contact e-mail in Round 2 and was only open for 1 week. Participants were not offered the opportunity to contribute further priority suggestions; therefore, this round focused on scoring the additional suggestions made in Round 2. Responses were scored using the same consensus criteria as in Round 2.

## Results

**Figure 1:**
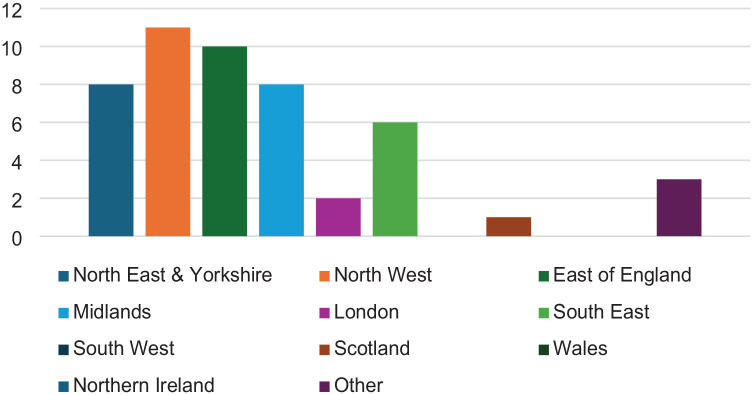
Geographical region of participants

**Figure 2:**
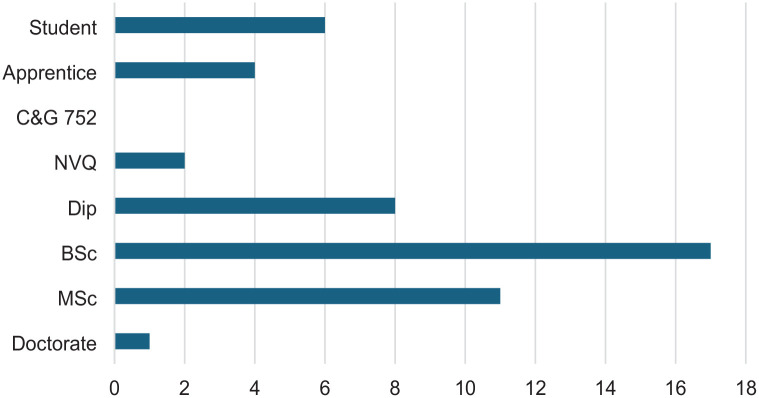
Highest qualification of participants

**Figure 3:**
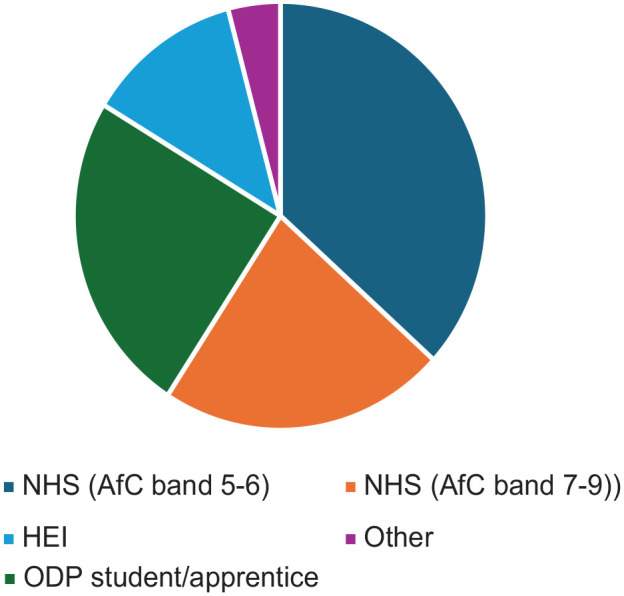
Primary employment of participants

Fifty-three responses were received in Round 1, with 49 deemed eligible (early demographic questions indicated some respondents were not ODPs). Participant demographics related to geographical areas, educational levels, and employment are presented in Figures 1–3. Forty-eight of the 49 participants provided contact details for participation in subsequent rounds. A total of 130 research priorities suggested by the participants (not all participants provided three topics) were subsequently coded, resulting in 62 research priorities. These were grouped into 12 provisional themes to improve the structural design for Round 2. Twenty-one participants completed scoring research priorities in Round 2, all of whom provided contact details to participate in Round 3. Nine free-text responses were received suggesting further research. This led to two additional priorities for Round 3. Seventeen participants completed Round 3.

**Figure 4:**
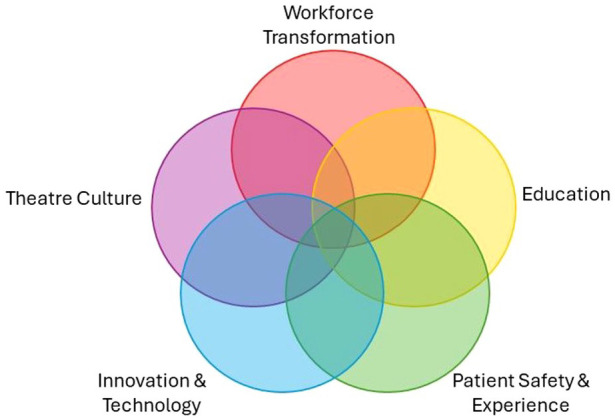
ODP research priority themes

Table 1 presents the priorities identified through Rounds 1 and 2, along with their scores for each test and a priority rank for those meeting consensus. In total, 31 research priorities were identified through consensus. Priority rank was determined first by mean score, followed by percentage agreement and coefficient of variance. Table 2 lists the top 10 priorities. Five key themes were identified from the research priorities meeting the consensus threshold (Figure 4). While some priorities and research questions fall under a single theme, the figure also indicates some overlap across thematic areas. Table 3 presents the current identified research priorities under the themes with which they most strongly align.

**Table 1:** Topics identified and scored for consensus as an ODP research priority, grouped by provisional theme

Topic	Mean	% agreement	CV (%)	Priority rank
**Professional profile and workforce**				
How do we raise the visibility of the profession? (public awareness)	4.48	85.6	16	2
How do we address student recruitment?	4.33	81.0	18	4
Factors affecting workforce retention and transience	4.29	90.5	22	6
Factors influencing job satisfaction	4.24	85.6	16	8
Development and impact of career pathways and progression	4.48	95.2	13	1
**Education**				
How do we maintain and enhance pre-registration education standards?	4.00	71.4	19	
Factors affecting and strategies to address student attrition	4.10	71.4	20	
Effective support for students	4.05	85.6	18	24
How do we enhance/embed research education pre-registration?	3.71	57.2	22	
How do we develop interprofessional education with students?	3.81	57.2	26	
Placement capacity – factors affecting, strategies to improve	4.19	71.4	23	
Developing and maintaining post-registration education and competency	4.14	90.5	17	14
Post-registration exposure to research opportunities	4.00	71.4	22	
The role of simulation-based learning methods, OSCEs, VR	4.05	81	19	25
**Scope of practice**				
Development and impact of advanced practice	4.00	81	26	29
Development and impact of enhanced practice	4.00	85.6	24	27
Development and impact of roles in ICU	4.14	85.6	23	18
Development and impact of roles in emergency departments	4.19	90.5	22	11
Development and impact of roles onwards	3.24	52.4	34	
Development and impact of roles – other outside areas	4.00	71.4	22	
Factors affecting/impact of obtaining prescribing rights	4.33	85.6	23	3
The impact of staff that rotate roles versus those that do not – performance, competency, impact on care delivery	3.57	57.2	35	
Factors affecting/Development/Impact of role equality and opportunities compared to other professions	4.14	85.6	23	19
Impact of working in other clinical areas during COVID	3.52	57.2	33	
Development and impact of critical care practitioners	3.90	71.4	27	
Research capacity	3.81	57.2	21	
Evaluating and developing level of autonomy	3.62	61.9	26	
**Patient safety**				
Patient safety	4.24	76.2	19	9
Never events	4.05	81	26	26
Techniques/Strategies for reducing infection	3.52	52.4	30	
Enablers and barriers to completing the WHO checklist	3.86	76.2	26	
**Patient care**				
Factors affecting quality of care	3.95	71.4	25	
Are there any differences in care by ODPs vs other professions?	3.81	61.9	25	
Patient experience	4.00	76.2	20	31
Patient outcomes	3.90	71.4	21	
Emergency interventions	3.95	71.4	25	
Quality improvement ideas	3.81	71.4	22	
Perioperative medicine development	3.71	57.2	26	
Impact of obesity	3.43	61.9	31	
**Anaesthetics**				
The ODP role in airway management	4.19	81	24	12
Efficacy of intraoperative analgesia – an ODP perspective	3.86	66.7	28	
**Theatre culture**				
The impact of perioperative culture on patient outcomes	4.10	85.6	18	21
Professional identity in the perioperative environment	4.0	71.4	22	
Promoting an inclusive research culture in the perioperative environment	4.15	76.2	18	13
Supporting ODPs to speak out when doing the right thing is harder than the wrong thing	4.08	76.2	29	23
Developing a psychologically safe environment in the operating theatre	4.29	81.0	21	7
**Communication, collaboration, and interprofessional working**				
The hidden profession: how do we educate others on the role of the ODP?	4.33	81.0	21	5
Communication in the perioperative environment: how does it impact staff and/or patient experience?	4.14	90.5	17	14
Investigate the influence of tacit knowledge on an ODP’s ability to make decisions	4.14	85.6	15	16
Leadership	4.14	81.0	20	20
**Human factors**				
An ODP’s experience of human factors	4.10	85.6	18	21
The impact of team working in the theatre environment	4.19	90.5	17	10
Systems focused resilience	3.86	71.4	20	
**Staff health and wellbeing**				
Health and wellbeing	3.75	57.2	28	
Factors which impact ODP morale/burnout/mental health in the perioperative environment	4.0	81.0	26	29
Waste gases: the effects of occupational exposure for ODPs	3.67	66.7	28	
Work-related MSK injuries in the ODP profession	3.81	66.7	28	
**Innovation and technology**				
How are ODPs involved in clinical innovations?	4.14	85.6	15	16
The use of AI in theatres: an ODP’s perceptions and involvement	3.86	71.4	23	
Development and implementation of education related to surgical technology	3.90	76.2	16	
Innovation of equipment and devices – how do theatre departments introduce new equipment and devices?	3.67	66.7	26	
**Other**				
The ‘green’ ODP: the contribution of ODPs to a sustainable environment	4.0	85.6	24	27
**Additional topics scored in Round 3**				
Impact/Value/Exploration of ODP involvement in pre-hospital care	3.76	70.6	34	
WHO debrief – barriers and enablers, impact on learning	4.0	70.6	19	

**Table 2:** Top 10 research priority topics

Rank	Topic
1	Development and impact of career pathways and progression
2	How do we raise the visibility of the profession? (public awareness)
3	Factors affecting/impact of obtaining prescribing rights
4	How do we address student recruitment?
5	The hidden profession: how do we educate others on the role of the ODP?
6	Factors affecting workforce retention and transience
7	Developing a psychologically safe environment in the operating theatre
8	Factors influencing job satisfaction
9	Patient safety
10	The impact of team working in the theatre environment

**Table 3:** Identified research priorities aligned to themes

Workforce transformation	Education	Patient safety and experience	Innovation and technology	Theatre culture
Development and impact of career pathways and progression (1)	How do we address student recruitment? **(4)**	Patient safety **(9)**	How are ODPs involved in clinical innovations? **(=** **16)**	Developing a psychologically safe environment in the operating theatre **(7)**
How do we raise the visibility of the profession? (public awareness) (2)	The hidden professions: how do we educate others on the role of the ODP? **(5)**	Never events **(26)**	The ‘green’ ODP: the contribution of ODPs to a sustainable environment **(=** **27)**	The impact of team working in the theatre environment **(10)**
Factors affecting/impact of obtaining prescribing rights (3)	Developing and maintaining post-registration education and competency **(=** **14)**	Patient experience **(31)**		Promoting and inclusive research culture in the perioperative environment **(13)**
Factors affecting workforce retention and transience (6)	Effective support for students **(24)**			Communication in the perioperative environment: how does it impact staff and/or patient experience? **(=** **14)**
Factors influencing job satisfaction (8)	The role of simulation-based learning methods, OSCEs, VR **(25)**			Investigate the influence of tacit knowledge on an ODP’s ability to make decisions **(=** **16)**
Development and impact of roles in emergency departments (11)				Leadership **(20)**
ODP’s role in airway management (12)				An ODP’s experience of human factors **(=** **21)**
Development and impact of roles in ICU (18)				The impact of perioperative culture on patient outcomes **(=** **21)**
Factors affecting/Development/Impact of role equality and opportunities compared to other professions (19)				Supporting ODPs to speak out when doing the right thing is harder than the wrong thing **(23)**
Development and impact of enhanced practice (= 27)				
Development and impact of advanced practice (= 29)				
Factors which impact ODP morale/burnout/mental health in the perioperative environment (= 29)				

Priorities that overlap themes are presented under the theme it most strongly aligns with. Priority ranking is included after priority.

## Discussion

Research is recognised as a core principle for both pre-and post-registration professional programmes. ODPs, as consumers of research, recognise that actively engaging in research benefits everyone, including service users, practitioners at all levels, teams, and organisations (Harris et al 2019). The CAHPR Shaping Better Practice through Research: Practitioner Framework details the need to make research core business; a concept echoed by NIHR and the Royal College of Physicians, who describe research ‘as everybody’s business’ (Royal College of Physicians 2022), recognise that all healthcare professionals can support research. Comer et al (2022) identified challenges in integrating research into AHP roles due to the research skills and support offered at the team level. Individuals perceive themselves as having adequate research skills; however, utilising these skills in clinical practice is hindered by a lack of support. This aligns with Conway et al (2026), who identified departmental support as a barrier to integrating research into practice. Establishing ODP research priorities can help identify where ODPs can become more involved in research opportunities within clinical practice and foster collaboration with professions with shared goals. The Association of Anaesthetists (2024) states that their research strategy focuses on patient safety, innovation, clinical outcomes, education and training, wellbeing, environment, sustainability, audits, and quality/service improvement projects. Many of these priorities overlap with those identified in this study. Therefore, these professions should collaborate.

Considering the top 10 research priorities identified in this study, most fall within the theme of workforce transformation. This is most likely attributable to ODPs’ position as a vulnerable profession, one with low public visibility, continuous obstacles in terms of career progression, and subsequent difficulties attracting candidates into training programmes and, therefore, the workforce. It may also reflect the transition to a degree as the threshold qualification, thus creating an increased desire for career progression and opportunities to maintain parity with counterparts in other healthcare professions. Therefore, priorities exploring how these issues can be overcome were at the forefront of most respondents’ views, achieving higher consensus. It will be interesting to see how this changes over time as work to address such issues is gradually undertaken and new career pathways become more widely available.

Across all priorities, there was a distinct lack of focus on patient care. This led the authors to reflect on how the question had been phrased. There were limited research priorities considering ‘the patient’; nor did the participants identify it as a missing priority in Round 2. Subsequently, only two priorities from the provisional themes of ‘Patient safety’ and ‘Patient care’ met consensus, with ‘Patient safety’ ranking within the top 10 and ‘Patient experience’ being the lowest-ranked topic. The majority of priorities submitted in this study focused on aspects related specifically to ODP as a profession, with little focus on the care delivered by the interprofessional perioperative team or the numerous contemporary issues relating to patient care. This warrants further exploration. Anecdotally, there is a lack of engagement and ownership in research on patient care, outcomes, and new techniques by ODPs, requiring further consideration of how ODPs perceive themselves in terms of autonomy, clinical decision-making, and care planning.

The authors were left to contemplate whether ODPs contribute to clinical research and whether clinical research in the perioperative environment is inclusive of all professions. This highlights the perception that research influencing surgical care is the responsibility of surgeons, and research relating to anaesthetic provision is the responsibility of anaesthetists; however, ODPs could contribute significantly to these aspects of care. The authors question whether ODPs are encouraged, supported, or recognised by such professions as potential contributors to research, or whether ODPs feel they have the capacity and capability to contribute. Comer et al (2022) suggested there is support for AHPs (including ODP) to undertake research at individual and organisational levels; however, this does not transcend to the team level. Therefore, embedding research into ODP clinical roles is a barrier, despite the relationship between research and positive patient outcomes.

A positive ODP research culture will promote the importance of ODPs, contributing new knowledge to the existing evidence repository. Engaging in research at all professional levels promotes the concept that care and education delivered in the perioperative environment and beyond are based on the best available evidence.

## Limitations and strengths

The Delphi method commonly convenes an ‘expert’ panel for the subject being explored (Iqbal & Pipon-Young 2009). However, we chose not to utilise this approach given the lack of ODPs with research expertise, as evidenced by the lack of published research by ODPs (Nightingale 2019). By offering an open opportunity to all ODPs, we hoped to encourage interaction and engagement with the work currently underway to enhance the research culture and offer opportunities for everyone’s voices to be heard. Research priorities should reflect the profession as a whole, and not just those considered the most actively engaged in research. Therefore, although this approach is considered a limitation, it is also a strength. Expertise is determined in many ways, and expertise by experience (of being an ODP) was considered a credible definition.

Another limitation was the number of participants. While there is no standard number, it typically varies from ten to 100 in published studies, with 30–50 considered optimum (Nasa et al 2021). The attrition from Round 1 to Round 2 was greater than anticipated and undoubtedly impacted analysis. The most noticeable impact is the reduced variation between calculated values, with several topics scoring the same on all consensus criteria, thus leading to joint priorities when determining rankings. Given the nature of the quasi-anonymous approach used, demographic information was only collected for Round 1. Tracing this information throughout subsequent rounds would have been useful for observing differences between participant responses in each round. Additional demographic information related to equality, diversity, and inclusivity may have also been useful in considering representation among participants. We recommend future research embed equality, diversity and inclusivity where appropriate.

Another limitation identified from Round 1 is confusion regarding what a ‘research priority’ is or how to clearly articulate one. This made interpretation and analysis challenging; responses ranged from more ‘political’ priorities for the profession to vague, single-word answers, where it was impossible to determine what exactly the suggested priority was, for example, ‘awareness’.

These results provide an initial structure outlining the research priorities and themes for the ODP profession. This advances ODP closer to aligning with its AHP counterparts and begins to address the R&D needs outlined in the ODP workforce report (HEE 2022). For ODPs wanting to undertake research, this study provides inspiration to address nationally recognised research priorities and themes. Consequently, research priorities aligned with national requirements offer a robust justification for accessing funding opportunities. This study provides clear research priorities, some of which may not be unique to ODP but correspond to and reflect other AHP professions (Society of Radiographers [SoR] 2017). This enhances the prospects for inter-professional work and accelerates collaborative research opportunities. The authors anticipate the CODP will adopt these findings and formalise the research priorities to optimise the outlined benefits.

## Conclusion

There is a professional requirement for all ODPs to contribute to research corresponding with their knowledge, skills, and experience. In addition to professional and regulatory requirements, there is a national drive to enhance the capacity and capability of AHP research. For ODPs to have a clear vision and embed research into their professional culture, it is important that professional stakeholders set a direction. This Delphi Study enabled ODPs to co-create the profession’s research priorities. By reaching consensus, ODPs now have several themes that will contribute to ODP professional development and patient care, and support the potential for nationally recognised funding opportunities. The individual priorities identified under each theme guide where priority interests currently lie, and are not intended as an exhaustive list. These are expected to change over time, although we anticipate the priority themes will remain the same. Overall, five key research themes have been established for the ODP profession: Workforce Transformation, Education, Patient Safety and Experience, Innovation and Technology, and Theatre Culture.
